# Supplementary material for: Legacy Effects of Early β-Adrenergic Stimulation Program Adipose Plasticity and Confer Metabolic Resilience in Obesity
Source: bioRxiv. 2026 Jun 29:2026.06.23.734002. Preprint. [Version 1] doi: 10.64898/2026.06.23.734002 (PMC13344982; doi:10.64898/2026.06.23.734002)
Supplement: Supplement 2 [file NIHPP2026.06.23.734002v1-supplement-2.pdf]

bioRxiv preprint doi: https://doi.org/10.64898/2026.06.23.734002; this version posted June 29, 2026. The copyright holder for this preprint (which was not certified by peer review) is the author/funder, who has granted bioRxiv a license to display the preprint in perpetuity. It is made available under a CC-BY 4.0 International license.

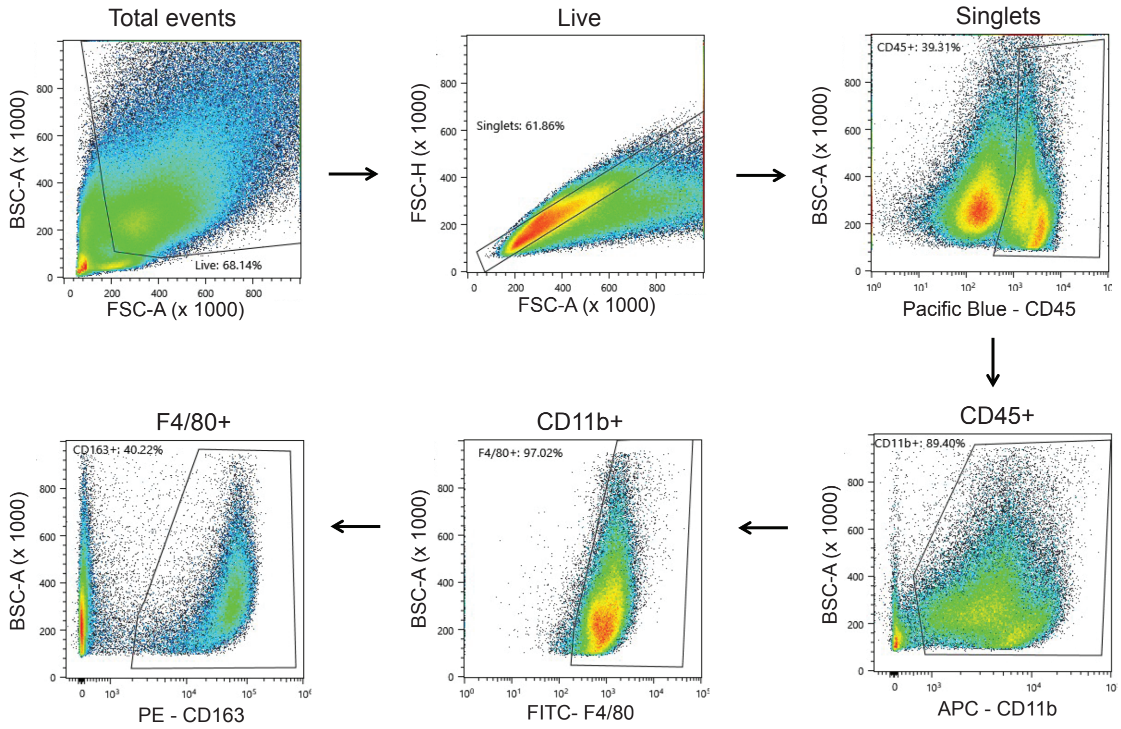

Supplementary Figure 1

bioRxiv preprint doi: https://doi.org/10.64898/2026.06.23.734002; this version posted June 29, 2026. The copyright holder for this preprint (which was not certified by peer review) is the author/funder, who has granted bioRxiv a license to display the preprint in perpetuity. It is made available under a CC-BY 4.0 International license.

Supplementary Figure 1: Flow cytometry gating strategy for the identification and quantification of adipose tissue macrophages (ATMs). (Related to Extended Data Figure 3).

Live cells/single cells were gated from the stromal vascular fraction of collagenase-digested eWAT. Hematopoietic lineage cells (CD45+) were selected from live/single cells of the stromal vascular fraction of collagenase digested eWAT. Then total ATMs were identified from the CD45+ cells based on CD11b and F4/80 expression (CD45+ CD11b+ F4/80+). Perivascular macrophages were identified amongst ATMs based CD163 expression (CD163+).
